# Severe pulmonary valve insufficiency caused by transjugular cannulation of pulmonary artery for right ventricular assist device: diagnosis and surgical solution—a case report

**DOI:** 10.1093/jscr/rjad389

**Published:** 2023-07-03

**Authors:** Leonhard Wert, Pia Lanmüller, Sascha Ott, Johanna Mulzer, Christoph T Starck, Volkmar Falk, Evgenij V Potapov

**Affiliations:** Department of Cardiothoracic and Vascular Surgery, Deutsches Herzzentrum der Charité, Augustenburger Platz 1, 13353 Berlin, Germany; Department of Cardiothoracic and Vascular Surgery, Deutsches Herzzentrum der Charité, Augustenburger Platz 1, 13353 Berlin, Germany; Department of Cardiac Anesthesiology and Intensive Care Medicine, Deutsches Herzzentrum der Charité, Augustenburger Platz 1, 13353 Berlin, Germany; Department of Cardiothoracic and Vascular Surgery, Deutsches Herzzentrum der Charité, Augustenburger Platz 1, 13353 Berlin, Germany; Department of Cardiothoracic and Vascular Surgery, Deutsches Herzzentrum der Charité, Augustenburger Platz 1, 13353 Berlin, Germany; DZHK (German Center for Cardiovascular Research), Berlin, Germany; Department of Cardiothoracic and Vascular Surgery, Deutsches Herzzentrum der Charité, Augustenburger Platz 1, 13353 Berlin, Germany; DZHK (German Center for Cardiovascular Research), Berlin, Germany; Charité – Universitätsmedizin Berlin, corporate member of Freie Universität Berlin and Humboldt-Universität zu Berlin, Charitéplatz 1, 10117 Berlin, Germany; Department of Health Sciences and Technology, ETH Zurich, Zurich, Switzerland; Department of Cardiothoracic and Vascular Surgery, Deutsches Herzzentrum der Charité, Augustenburger Platz 1, 13353 Berlin, Germany; DZHK (German Center for Cardiovascular Research), Berlin, Germany

## Abstract

Implantation of a temporary percutaneous right ventricular assist device (RVAD) in patients with right heart failure after left ventricular assist device (LVAD) implantation is an established technique that may cause complications. We present a 60-year-old male patient who underwent urgent LVAD implantation. On the second postoperative day the patient developed acute right heart failure. We implanted a temporary percutaneous RVAD with two cannulas via the right internal jugular vein and the right femoral vein. Transesophageal echocardiography revealed severe pulmonary insufficiency. After performing re-sternotomy we anastomosed a prosthetic graft to the pulmonary trunk (PT), performed subxiphoid tunneling of the graft and replaced the transjugular outflow cannula. The pulmonary regurgitation caused by the percutaneous transvalvular cannula disappeared. In such case a direct anastomosis to the PT is the solution.

## INTRODUCTION

Right heart failure after left ventricular assist device (LVAD) implantation presents a major challenge. Early right heart failure affects up to 15% of patients post LVAD implantation [1]. Mechanical support of the right ventricle is considered mandatory. Implantation of a temporary percutaneous right ventricular assist device (RVAD) with a centrifugal pump is an established technique to treat postoperative right heart failure in this setting [2, 3].

## CASE PRESENTATION

A 60-year-old male patient (172 cm, 70 kg, body mass index 23.7 kg/m^2^) with a history of ischemic cardiomyopathy, first diagnosed 23 months ago, was admitted with acute on chronic heart failure. He presented with progressive dyspnea for 2 days and persistent nausea that was linked to a history of chronic gastritis. The patient’s history included paroxysmal atrial fibrillation under oral anticoagulation with rivaroxaban, as well as arterial hypertension, chronic kidney disease and hepatomegaly. Coronary artery disease had been treated with percutaneous transluminal coronary angioplasty and drug-eluting stents and currently required no intervention.

**Figure 1:**
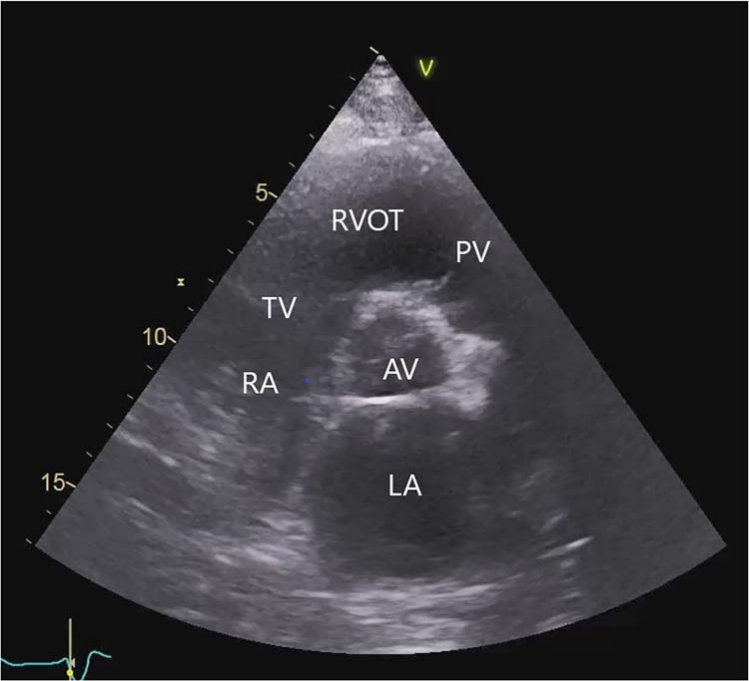
Transthoracic echocardiogram before LVAD implantation. Parasternal short-axis view. AV (aortic valve), LA (left atrium), PV (pulmonary valve), RA (right atrium), RVOT (right ventricular outflow tract), TV (tricuspid valve).

The transthoracic echocardiogram revealed severe dilatation of the left ventricle with a left ventricular end-diastolic diameter of 7.4 cm and a left ventricular systolic function of 15–20% (using Simpson’s biplane method of disks). The size of the right ventricle was normal. The right ventricular systolic function was mildly reduced by visual estimation. There was moderate to severe mitral regurgitation. The aortic valve was normal. The pulmonary and tricuspid valves showed trivial regurgitation (Fig. 1, Video S1 in the Supplementary Material online).

While inotropic support continuously declined, the decision was made to proceed with urgent LVAD implantation (HeartWare, HVAD) via full median sternotomy using an off-pump technique. Intraoperative transesophageal echocardiography confirmed a correct position of the inflow cannula and a moderately reduced right ventricular function with moderate tricuspid regurgitation despite the use of nitric oxide ventilation and continuous application of inotropes.

Initially inotropes could be discontinued and nitric oxide ventilation tapered adequately. The patient was extubated 6 h after arrival at the intensive care unit.

**Figure 2:**
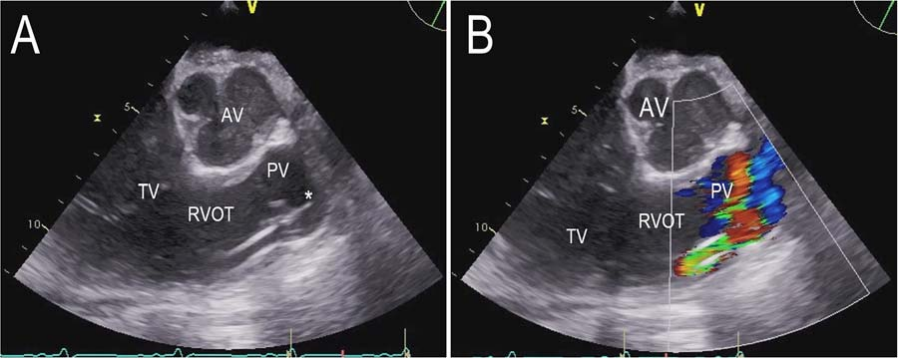
Transthoracic echocardiogram after implantation of a percutaneous RVAD with two cannulas via the right IJV and right FV. Parasternal long-axis view demonstrating the cannula position in the pulmonary valve (**A**). Color Doppler transthoracic echocardiogram after implantation of a percutaneous RVAD with two cannulas via the right IJV and right FV. Parasternal long-axis view demonstrating severe pulmonary regurgitation due to cannula position in the pulmonary valve (**B**). AV (aortic valve), PV (pulmonary valve), RVOT (right ventricular outflow tract), TV (tricuspid valve), ^*^ tip of cannula.

On the second postoperative day the patient showed hemodynamic impairment with signs of right ventricular failure. A temporary percutaneous RVAD was implanted with two cannulas via the right internal jugular vein (IJV) and the right femoral vein (FV). Despite RV support, LVAD flow did not increase and hemodynamic instability failed to improve. Transthoracic echocardiography revealed severe pulmonary insufficiency caused by the cannula of the RVAD. The blood recirculated in the congested right ventricle (Fig. 2, Videos S2 and S3 in the Supplementary Material online).

**Figure 3:**
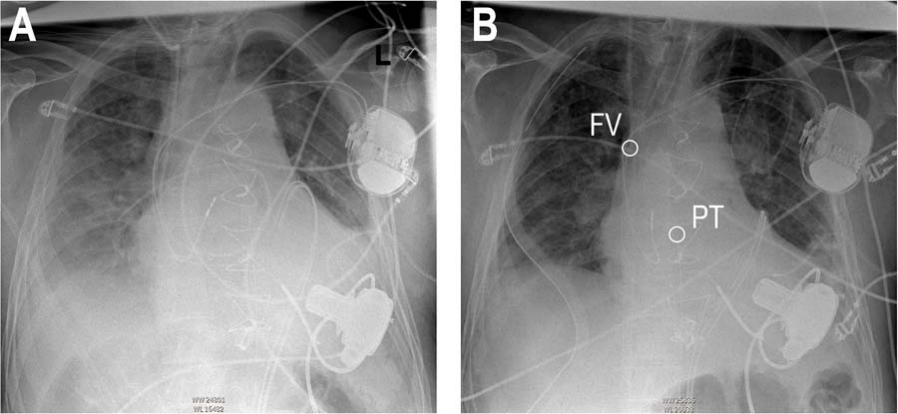
Anteroposterior thoracic radiography after LVAD implantation (**A**). Anteroposterior thoracic radiography after RVAD cannulation of the PT (**B**). Tip of cannula in the right FV, tip of cannula in the PT.

**Figure 4:**
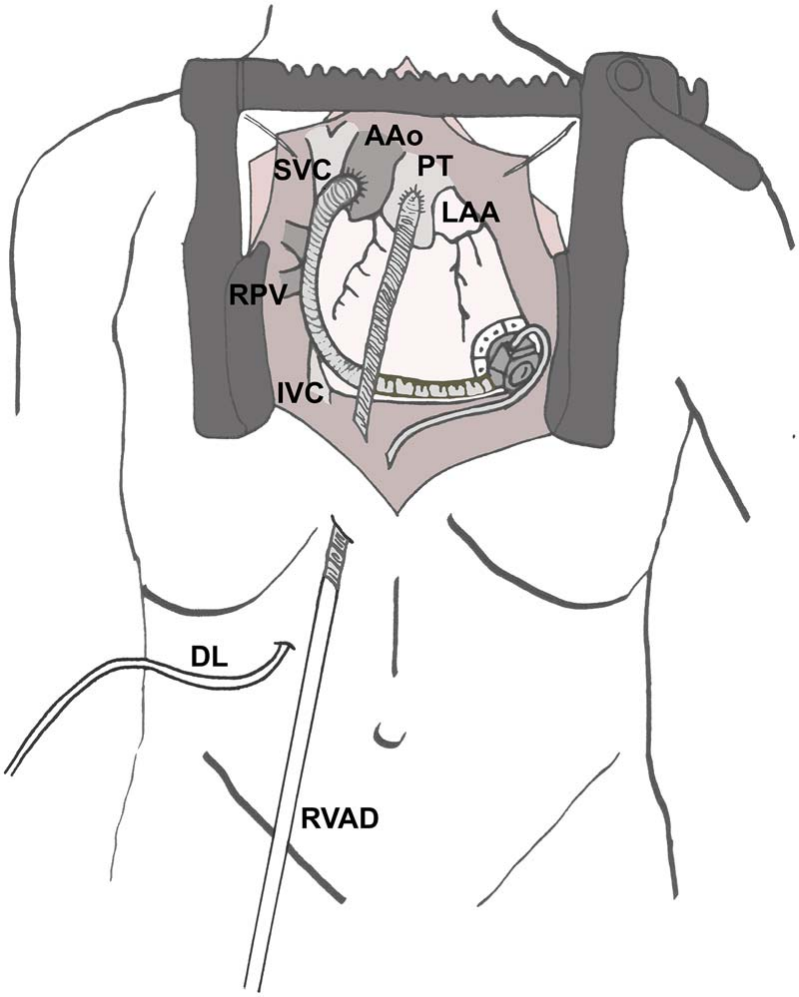
Surgical site after implantation of the cannula in the PT. AAo (ascending aorta), DL (driveline of left ventricular assist device), IVC (inferior caval vein), LAA (left atrial appendage), PT (pulmonary trunk), RPV (right pulmonary veins), RVAD (cannula of right ventricular assist device) SVC (superior caval vein).

Re-sternotomy was performed. The pulmonary trunk (PT) was clamped. A 10-mm prosthetic polyester graft was anastomosed to the PT. After subxiphoid tunneling of the graft the transjugular outflow cannula was replaced by a cannula in the anastomosed graft. The cannula in the right FV remained in position in the right atrium (Figs 3 and 4). The hemodynamic situation with LVAD and RVAD flow improved immediately.

After 23 days of RVAD support both cannulas were explanted. The cannula in the FV was only punctured, allowing pull it out simply. The subxiphoid tunneled graft was stretched, ligated and cut very short to allow it to retract into the thorax.

The 1-year postoperative control echocardiography confirmed a normal pulmonary valve and a regular size and function of the right ventricle (Video S4 in the Supplementary Material online).

In conclusion, the pulmonary artery cannula of the percutaneous RVAD may cause severe pulmonary insufficiency. In such case, a direct anastomosis to the PT is the solution.

## Supplementary Material

video_s1_rjad389

video_s2_rjad389

video_s3_rjad389

video_s4_rjad389

## Data Availability

Data is available upon request.
